# Ovarian cancer classification based on dimensionality reduction for SELDI-TOF data

**DOI:** 10.1186/1471-2105-11-109

**Published:** 2010-02-27

**Authors:** Kai-Lin Tang, Tong-Hua Li, Wen-Wei Xiong, Kai Chen

**Affiliations:** 1 Shanghai Center for Bioinformation Technology, Shanghai, 200235, China; 2 Department of Chemistry, Tongji University, Shanghai, 200092, China

## Abstract

**Background:**

Recent advances in proteomics technologies such as SELDI-TOF mass spectrometry has shown promise in the detection of early stage cancers. However, dimensionality reduction and classification are considerable challenges in statistical machine learning. We therefore propose a novel approach for dimensionality reduction and tested it using published high-resolution SELDI-TOF data for ovarian cancer.

**Results:**

We propose a method based on statistical moments to reduce feature dimensions. After refining and *t*-testing, SELDI-TOF data are divided into several intervals. Four statistical moments (mean, variance, skewness and kurtosis) are calculated for each interval and are used as representative variables. The high dimensionality of the data can thus be rapidly reduced. To improve efficiency and classification performance, the data are further used in kernel PLS models. The method achieved average sensitivity of 0.9950, specificity of 0.9916, accuracy of 0.9935 and a correlation coefficient of 0.9869 for 100 five-fold cross validations. Furthermore, only one control was misclassified in leave-one-out cross validation.

**Conclusion:**

The proposed method is suitable for analyzing high-throughput proteomics data.

## Background

Recent advances in proteomics technologies have enabled large-scale analysis of complex protein expression patterns, protein-protein interactions and posttranslational modifications. One of these technologies, surface-enhanced laser desorption/ionization time-of-flight (SELDI-TOF) mass spectrometry has shown great potential in early disease diagnosis and prevention [1,2]. The majority of studies on SELDI-TOF analysis have focused on the classification problem. The potential efficacy of SELDI-TOF serum protein profiling for cancer classification has been demonstrated by the identification of biomarkers for ovarian cancer [3-6], breast cancer[7], prostate cancer[8], liver cancer[9], lung cancer[10].

Data sets are obtained for biological samples collected from different patients classified in different classes (e.g. disease versus control, disease A versus disease B, successful versus unsuccessful treatment) for mass spectrometry data after sample fractionation under different physical conditions (so-called chip surfaces). The mass spectrometer typically provides signal intensities in a range of *m*/*z *ratios up to 20,000 Da. This leads to a vector of 5000-20,000 numerical values for each mass spectrum. In practice, for a given patient these data can be obtained for a sample pre-processed on several different chip surfaces for several replicates, thus potentially leading to over 100,000 numerical variables per patient. Although the number of variables can be very high in these applications, the number of patients and samples is typically rather small. This leads to rather untypical pattern problems in which the number of input variables is several orders of magnitude higher than the number of samples.

Although significant progress has been made in proteomics technologies, the development of tools for the analysis and interpretation of the large amounts of data produced remains a challenge. Some methods have been reported for class discrimination based on SELDI mass spectra [11,12]. Vlahou *et al*. [13] tested the classification and regression tree (CART) method for discrimination of ovarian cancer from benign diseases and healthy controls and achieved a cross-validation accuracy of 81.5%. Another approach was via tree-based methods [14]. Li *et al*. [15] applied the GA/KNN method to SELDI proteomics data analysis and achieved 95% accuracy (range 90-100%) for unaffected specimens and 98% (range 90-100%) for cancer specimens. Purohit *et al*. [16] combined information from multiple multivariate models to accurately classify type-diabetes and control subjects, with 88.9% specificity and 90.0% sensitivity. Zhang *et al*. [17] developed a recursive support vector machine (R-SVM) algorithm to select important genes and biomarkers for the classification of noisy data and used it for mass spectrometry and microarray data.

SELDI-TOF mass spectrometry data also consist of tens of thousands of *m/z *ratios per specimen and an intensity level for each *m/z *ratio. Thus, dimensionality reduction is a critical stage before discrimination using such data. Traditionally, there are two types of methods used to reduce dimensionality. One is variable selection and the other is variable transformation such as linear or nonlinear combination of variables. Variable selection techniques do not alter the original representation of the variables, but merely select a subset of variables derived from the large set of profiles. A classifier is then built using the reduced input set. In the context of classification, feature selection techniques can be organized into three categories, depending on how they combine the feature selection search with the construction of the classification model: filter methods, wrapper methods and embedded methods [18]. Variable transformation methods are used to construct a small set of aggregate variables such that every variable combines many inputs in the profile, including methods based on projection or compression such as principal component analysis and kernel transformation. Regardless of the type of method adopted, reduction of high dimensionality to an appropriate low dimension can substantially influence the results. Yu *et al*. [19] developed a four-step strategy for data preprocessing and achieved average sensitivity of 97.38% and specificity of 93.30%. Hauskrecht *et al*. [20] used univariate feature selection strategies with a heuristic based on multivariate decorrelation filtering to improve the classification performance for a pancreatic cancer data set. Bhanot *et al*. [21] proposed a novel method for phenotype identification involving stringent noise analysis and a filtering procedure combined with the results of several machine learning tools to produce a robust predictor. They were able to distinguish cancer from non-cancer cases with sensitivity of 90.31% and specificity of 98.81%. From a clinical viewpoint, such positive results demonstrate the potential of this new bioassay technology.

The majority of the above-mentioned studies on analysis of proteomics data have focused on one feature reduction approach. To improve the classification performance for ovarian cancer, we use a different method based on statistical moments to transform data. The aim of our method is not only to preserve the data properties, but also to reduce the variables. Four statistical moments (mean, variance, skewness and kurtosis) are used to describe the data to reduce the feature dimensions and extract the characteristics of the data at the same time. A kernel algorithm could be useful in mining high-dimensional SELDI-TOF data. We demonstrate the applicability of the kernel PLS (KPLS) to SELDI-TOF data analysis and show that the kernel is capable of reliable discrimination between cancer and healthy samples. Dimensionality reduction and classification can be carried out simultaneously using KPLS. Good results were obtained and only one control was misclassified in the leave-one-out cross validation.

## Methods

### Data preprocessing

In general, MS data are characterized by small numbers of very high-dimensional samples. There are potentially tens of thousands of intact and cleaved proteins in the human serum proteome, so finding a single elusive disease-related protein is very tedious, requiring the laborious separation and identification of every biomarker.

For the raw high-resolution SELDI-TOF ovarian data set provided by the National Cancer Institute (available at http://home.ccr.cancer.gov/ncifdaproteomics/ppatterns.asp), the data can be written as *S *= {(*x*_*i *_, *y*_*i*_) ∈ *x*_*i *_∈ R^m^, *y*_*i *_= ± 1, *i *= 1, 2, ..., *n*}, where *x*_*i *_is an intensity vector according to a sorted sequence of *m*/*z *ratios and *y*_*i *_is the class label of *x*_*i *_(âˆ’1 for non-cancer, +1 for cancer). When the feature space has high dimensionality, feature selection and transformation become crucial as the first step towards pattern recognition. For the raw high-resolution SELDI-TOF ovarian data set for 95 control samples and 121 cancer samples, the dimensionality of the original feature space is >370,000.

In the present study, the data were preprocessed using three steps, (1) refining (2) *t*-testing and (3) statistical transformation. All the procedures are independent of the particular classifier that will be used later.

### Refining

The data are aligned according to the sorted union of *m*/*z *values into an intensity frame with missing data. The data rows represent *m*/*z *values and columns represent samples or observations. Any *m*/*z *values that do not have certain desired properties are removed from the data set, thus excluding *m*/*z *values that have missing values in one of the columns. Then the data are refined and the missing data are ignored. The dimensionality is thus reduced to 39,905. This is defined as set A.

### *t*-Testing

For each *m*/*z *ratio, we compare the distribution of the data using a two-sided *t*-test (the null hypothesis H_0 _is that cancer and non-cancer have the same distribution) at a significance level of 1%. The data obtained are defined as set B. The dimensionality of the feature space is reduced from 39,905 to 24,545.

**Figure 1:**
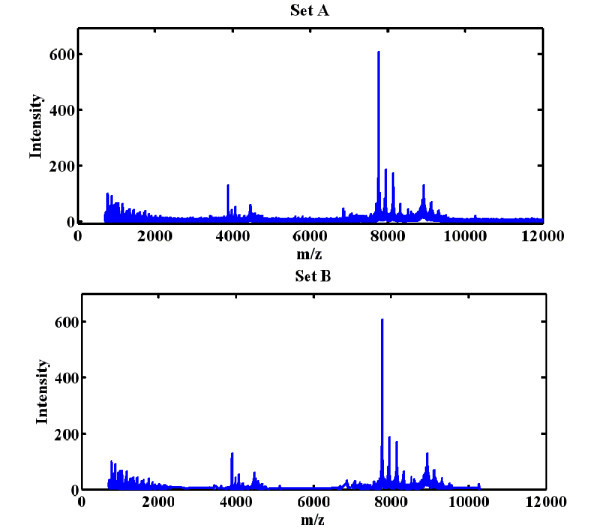
**Mass spectra for set A (top) and set B (bottom)**. A mass spectrum for serum from a cancer patient, with *m*/*z *values on the horizontal axis and intensity values indicating the relative ion abundance on the vertical axis.

Figure 1 depicts a mass spectrum for serum from a cancer patient. Although the spectrum of set B resembles that of set A, more than 15000 *m*/*z *values that are not significantly different between cancer and non-cancer have been filtered.

### Data transformation based on statistical moments

The mass data were first divided into several intervals. Waveform segments are better than individual *m*/*z *values in representing the characteristics of MS data. Thus, we selected variables that could represent the characteristics of each waveform segment. After several experiments, statistical moments were selected for further analysis.

The mean, variance, skewness and kurtosis were calculated for each interval. The mean is the sum of the observations divided by the number of observations, which describes the central location of the data. The variance is the expected value of the square of the deviation from its own mean. It can be expressed as the average of the square of the distance of each data point from the mean. The variance describes the spread of the data. A distribution, or data set, is symmetric if it looks the same to the left and right of the center point. The skewness is a measure of symmetry or, more precisely, a lack of symmetry. Kurtosis is a measure of whether the data are peaked or flat relative to a normal distribution. Data sets with high kurtosis tend to have a distinct peak near the mean, decline rather rapidly, and have heavy tails. Data sets with low kurtosis tend to have a flat top near the mean rather than a sharp peak.

**Figure 2:**
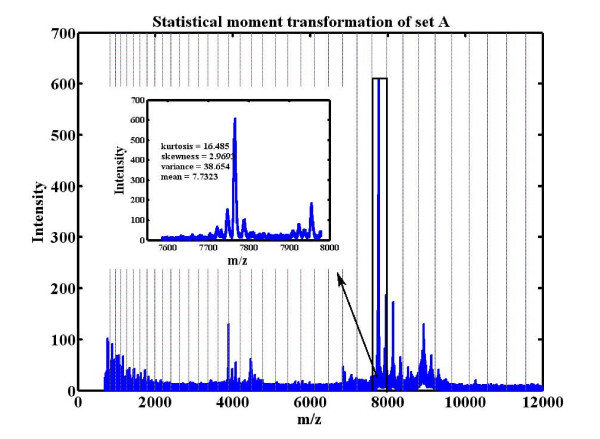
**Statistical moment transformation for set A**. After division of the data into several intervals, the statistical moments were calculated for one of the intervals (for example *m*/*z *7600-8000) and used as new variables to represent the characteristics of the spectrum.

For univariate data *Y*_*1*_, *Y*_*2*_, ..., *Y*_*N*_, the following formulas hold, where  is the mean, *S *is the standard deviation, and *N *is the number of data points:

The calculated statistical moments, which describe the characteristics of the spectrum from a statistical point of view, were then used as new variables for classification. Figure 2 depicts the statistical moment transformation for set A.

**Figure 3:**
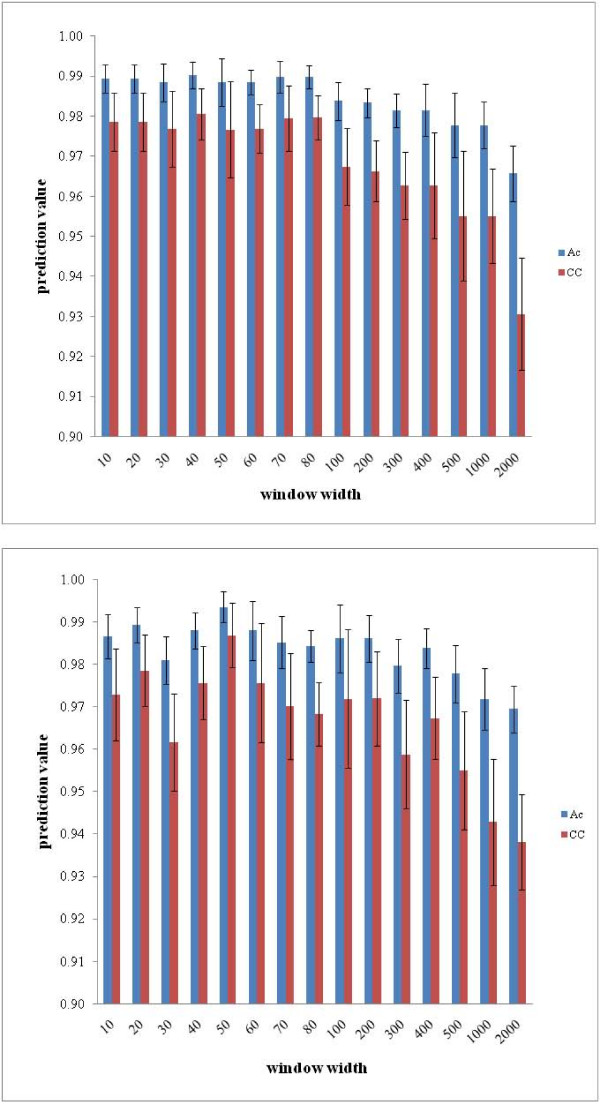
**Results for different window widths**. Ac/CC results for set A (top) and set B (bottom) for different window widths.

It is important to note that the results are influenced by the window width. The interval width was optimized based on modeling results. Figure 3 shows the results for different window widths. The best results were obtained for a width of 40 for set A and 50 for set B. Therefore, after transformation based on statistical moments, sets A and B were transformed to sets C and D, reducing the dimensionality to 3992 and 1964, respectively.

### Classifications by KPLS

SVM was originally proposed and developed by Vapnik [22] and has been applied in various areas. KPLS, like SVM, is a learning method based on a kernel function. Rosipal [23] introduced KPLS as a nonlinear extension to the linear PLS method [24] and KPLS is a powerful machine learning tool for classification and regression. KPLS comprises construction of a kernel matrix and PLS. The difference between KPLS and traditional PLS is that XX^T ^is replaced by a kernel matrix. Therefore, the relationships among variables are replaced by those among samples.

The fundamental principle of a kernel is the feature space *F *(φ: *x*_*i *_∈ R^N ^→ Φ (*x*_*i*_) ∈ F) in which the original data items are embedded [23]. In this space linear relations are explored among images of the data items. However, these feature images φ(*x*_*i*_) are not necessary and are also impossible to obtain. Thus, pairwise inner products are computed from the original data items using kernel function to reveal the relations implicitly.

Given a set of vectors X = (x_1, ... *n*_), where *n *denotes the number of samples and κ denotes kernel function; K is defined as the kernel matrix whose entries are generated by such pattern:

The training kernel matrix is symmetric and square, whereas the testing kernel matrix is usually not because the vectors involved in computing the inner product are separate from the training set and testing set.

To centralize the mapped data in a feature space *F *we can simply apply the following procedures [25]:

where *I *is an n-dimensional identity matrix and *1*_*n*_, *1*_*nt *_represent the vectors whose elements are 1 of length *n *and *n*_*t*_, respectively. *K*_*t *_is the (*n*_*t *_× *n*) "test" matrix whose elements are *K*_*ij *_= *K*(*x*_*i*_, *x*_*j*_), where  and  are the testing and training points, respectively.

Before applying KPLS, identification of a proper kernel function and optimal kernel parameters is crucial. Commonly used kernel functions include linear, polynomial, radial basis and sigmoid functions. Different kernel functions are selected for different problems. Currently, there is no systematic methodology for the selection of kernel functions. In the present study, experiments revealed that a polynomial kernel function was better than other functions:

The parameter *p *denotes the degree of the polynomial and was defined as *p *= 3 in experiments for the present study. The parameter *r *denotes the relative weighting of the different degree mononomials. Here *r *is fixed as 1 for simplicity under the assumption that all subsets corresponding to each mononomial make an equal contribution to the kernel.

## Results and discussions

Accuracy (Ac), sensitivity (Sn) and specificity (Sp) are often used to evaluate prediction systems. The correlation coefficient (CC), which ranges from -1 to +1, can also be used. The closer the CC value is to +1, the better is the prediction system. Sn, Sp, Ac and CC are expressed in terms of true positive (TP), false negative (FN), true negative (TN) and false positive (FP) rates:

Results for combinations of the four statistical moments are listed in Table 1. For each five-fold validation, the random experiment was repeated 100 times independently. The mean plays a very important role in classification. Poor results were obtained using skewness and kurtosis separately and the best results were obtained when the four statistical moments were all used for calculation.

**Table 1:** Five-fold cross validation of statistical transformation

	Mean	Variance	Skewness	Kurtosis	Ac	CC	Sn	Sp
Average	√				0.9917	0.9832	0.9860	0.9989
SD					0.0020	0.0039	0.0040	0.0033
Average		√			0.9639	0.9272	0.9587	0.9705
SD					0.0078	0.0156	0.0117	0.0067
Average			√		0.7796	0.5718	0.7231	0.8516
SD					0.0195	0.0371	0.0273	0.0201
Average				√	0.6986	0.3920	0.7124	0.6811
SD					0.0187	0.0370	0.0258	0.0238
Average	√	√			0.9907	0.9814	0.9851	0.9979
SD					0.0031	0.0062	0.0052	0.0044
Average	√		√		0.9921	0.9842	0.9868	0.9989
SD					0.0022	0.0045	0.0043	0.0033
Average	√			√	0.9898	0.9796	0.9826	0.9989
SD					0.0029	0.0059	0.0047	0.0033
Average		√	√		0.9625	0.9242	0.9595	0.9663
SD					0.0046	0.0092	0.0082	0.0067
Average		√		√	0.9569	0.9129	0.9562	0.9579
SD					0.0069	0.0140	0.0088	0.0122
Average			√	√	0.7722	0.5447	0.7587	0.7895
SD					0.0132	0.0277	0.0145	0.0253
Average	√	√	√		0.9921	0.9842	0.9876	0.9979
SD					0.0022	0.0045	0.0044	0.0044
Average	√	√		√	0.9921	0.9842	0.9876	0.9979
SD					0.0022	0.0045	0.0044	0.0044
Average	√		√	√	0.9917	0.9832	0.9868	0.9979
SD					0.0020	0.0039	0.0043	0.0044
Average		√	√	√	0.9903	0.9804	0.9843	0.9979
SD					0.0034	0.0069	0.0047	0.0044
Average	√	√	√	√	0.9935	0.9869	0.9950	0.9916
SD					0.0037	0.0075	0.0055	0.0042

Ac, accuracy; CC, correlation coefficient; Sn, sensitivity; Sp, specificity.

Tables 2, 3, 4 and 5 list the validation results for data sets A-D, respectively. The classification performance was assessed using five-fold cross-validation, five-fold proportional validation (randomly selecting 80% controls and 80% cancers as the training set and testing the classifier on the remaining samples) and leave-one-out cross-validation. For each five-fold validation, the random experiment was repeated 100 times independently. Comparison of the standard deviation for five-fold cross and proportional validations reveals that KPLS is stable for classification. KPLS avoids the SVM overfitting problem reported by Yu *et al*. [19]. The SVM results indicate that overfitting seems to be more serious for the control samples, whereas KPLS leads to good results for both control and cancer samples. Besides decreasing the computational complexity, the procedure cleans the original data and explores their category traits for classification. All four sets yield good classification results, with the best performance observed for set D in all validation tests. In leave-one-out cross-validation, only one control was misclassified.

**Table 2:** Validation for set A

	Ac	CC	Sn	Sp
Leave-one-out validation	0.9815	0.9627	0.9752	0.9895
Five-fold cross validation	0.9810	0.9618	0.9752	0.9884
SD	0.0046	0.0092	0.0067	0.0060
Five-fold proportional validation	0.9833	0.9663	0.9715	0.9923
SD	0.0101	0.0203	0.0172	0.0172

**Table 3:** Validation for set B

	Ac	CC	Sn	Sp
Leave-one-out validation	0.9907	0.9815	0.9833	1.0000
Five-fold cross validation	0.9704	0.9403	0.9636	0.9789
SD	0.0050	0.0100	0.0070	0.0050
Five-fold proportional validation	0.9829	0.9657	0.9787	0.9846
SD	0.0098	0.0201	0.0307	0.0211

**Table 4:** Validation for set C

	Ac	CC	Sn	Sp
Leave-one-out validation	0.9907	0.9814	1.0000	0.9835
Five-fold cross validation	0.9904	0.9811	0.9847	0.9977
SD	0.0145	0.0284	0.0241	0.0113
Five-fold proportional validation	0.9900	0.9800	0.9835	0.9979
SD	0.0144	0.0286	0.0254	0.0102

**Table 5:** Validation for set D

	Ac	CC	Sn	Sp
Leave-one-out validation	0.9954	0.9906	1.0000	0.9895
Five-fold cross validation	0.9935	0.9869	0.9950	0.9916
SD	0.0037	0.0075	0.0055	0.0042
Five-fold proportional validation	0.9909	0.9817	0.9937	0.9937
SD	0.0188	0.0376	0.0193	0.0193

In order to evaluate the applicability of the proposed method to other data sets, the high resolution pancreatic cancer premalignant data downloaded from National Cancer Institute http://home.ccr.cancer.gov/ncifdaproteomics/ppatterns.asp were classified by the method. The classification accuracy using seven different classifiers and three kinds of feature selection methods ranged from 0.5611 to 0.7500 [26]. The 10-fold cross-validation results in Table 6 (repeated 100 times independently) showed that the proposed preprocessing method performed well. The data using statistical moments preprocessing have better accuracies than those without preprocessing and those in reference, thus suggest the utility of the method in proteomics data analysis more generally.

**Table 6:** Results of pancreatic cancer premalignant data

		Ac	CC	Sn	Sp
Without preprocessing	Average	0.6917	0.3884	0.7075	0.6791
	stdev	0.0609	0.1257	0.1137	0.0861
After preprocessing	average	0.7697	0.5485	0.7008	0.8240
	stdev	0.0307	0.0611	0.1517	0.1152

The number of proteomics data samples is much less than the number of variables. Therefore, there are some difficulties in data analysis: useless variables interfere with the results and useful information is hidden. After data preprocessing to reduce the dimensionality, KPLS is used as a variable nonlinear recombination method in which the relationships among variables are replaced by those among samples. Thus, both samples and variables are considered in the classification and good results are obtained.

Statistic moments are applicable to many different domains, for example, aspects of image processing, ranging from invariant pattern recognition and image encoding to pose estimation. When applied to images, statistic moments describe the image content (or distribution) with respect to its axes. They are designed to capture both global and detailed geometric information about the image. Recently, a novel method, named statistical moment-based method, was proposed for structural damage detection, which was believed to be sensitive to local structural damage but insensitive to measurement noise [27]. Four statistical moments transformation yield novel variables and reduce dimension of feature space by reconstruct the data. In this paper, the result showed that the statistic moments can not only reduce the dimensionality of the feature space but also reflect the differences between the cancer and the healthy samples. Using the technology of reconstruction data, such as statistic moments, a novel insight will be created into large biological data and good performance will be achieved in other fields of biological statistics.

## Conclusions

Serum proteomic profiling is a new approach to cancer diagnosis. It confronts a challenging environment, as it combines measurement technologies that are new in the clinical setting with novel approaches to processing and interpreting high dimensional data. Statistical moments' transformation for feature reduction has been playing active roles in analyzing such data. By efficient preprocessing of high-resolution ovarian MS data, KPLS achieve a satisfied performance of classifying cancer and the healthy. On the one hand, data preprocessing reduces the dimensionality of feature space; on the other hand KPLS extrudes the most significant category traits for the coming classification. The proposed method is general enough that it can be adapted to other proteomics data such as MALDI-TOF data and even NMR metabolomics profiling.

## Authors' contributions

KLT performed the data analysis and wrote the manuscript, WWX evaluated the results. KC participated in the study design. THL conceived and supervised the study. All authors have read and approved the final manuscript.
